# Osteomyelitis complicating secondarily infected atopic eczema: two case reports and a narrative literature review

**DOI:** 10.1186/s12895-019-0098-0

**Published:** 2020-02-03

**Authors:** Josiah T. Masuka, Katherine Troisi, Zamambo Mkhize

**Affiliations:** 1 Department of Dermatology, Nelson R Mandela School of Medicine, Private Bag X7, Congella, Durban, 4013 South Africa; 2 Department of Dermatology, Edendale Hospital, 89 Selby Msimang Rd, Pleissislaer, Pietermaritzburg, 3201 South Africa; 3 Department of Orthopaedics, Edendale Hospital, 89 Selby Msimang Rd, Pleissislaer, Pietermaritzburg, 3201 South Africa

**Keywords:** Osteomyelitis, Osteo-arthritis, Atopic eczema, Secondary infection, *Staphylococcus aureus*

## Abstract

**Background:**

Atopic eczema is a relapsing, itchy chronic cutaneous inflammatory disease that commonly affects children. The disease is often complicated by cutaneous infections such as eczema herpeticum, eczema vaccinatum and a varied number of bacterial infections – impetigo, cellulitis and erysipelas. However, rare case reports of infective endocarditis, otitis media and osteo-articular infections have been associated with atopic eczema. These associations possibly represent the extracutaneous infectious complications of atopic eczema.

**Case presentation:**

Here we present two cases of osteomyelitis in HIV negative children with habitual scratching of poorly managed and/or uncontrolled atopic eczema respectively. Both cases presented to the orthopaedic surgeons and were admitted as acute phalangeal osteomyelitis and acute – on – chronic tibial osteomyelitis respectively. The first case was an 8 year old girl who had moderate-severe poorly-controlled atopic eczema and contiguously spread phalangeal osteomyelitis. The second case was an 11 year old pre-pubertal boy who had untreated atopic eczema and tibial osteomyelitis possibly from haematogenously spread *Staphylococcus aureus* infection. Both were successfully discharged from hospital and currently have well controlled eczema. The 11 year old patient is also being reviewed monthly by the orthopaedic surgeons and is chronic suppressive antibiotics. He may require sequestrectomy, should it be needed.

**Conclusions:**

Invasive staphylococcal and streptococcal osteo-articular (OA) infection can arise as an extra-cutaneous infectious complication of poorly controlled atopic eczema. It is more common in the 3 to 15 year age group and especially in boys with a septic arthritis to osteomyelitis ratio of around 29:5. Clinicians should maintain a high index of suspicion in patients with moderate-severe atopic eczema and they ought to promptly manage these OA infections with intravenous antibiotics to avoid further complications.

## Background

Osteomyelitis has rarely been associated with secondarily infected atopic eczema [1–4]. In addition, phalangeal and/or hand osteomyelitis has rarely been described in literature as contiguous contamination secondary infection in atopic eczema or on the hands [5, 6]. In one reported case series, three cases of acute phalangeal osteomyelitis due to habitual scratching in children with severely infected atopic eczema has been documented [5]. In all three children, no apparent indicators of haematogenous bacterial dissemination such as elevated erythrocyte sedimentation rate (ESR) or pyrexia were observed [7]. On the other hand, invasive staphylococcal bacteremia and haematogenous osteo-articular infections have also been reported sporadically in association with atopic eczema [3, 8, 9]. These cases represent some of the infrequently reported invasive complications of secondarily infected atopic eczema which require prompt and definitive management to prevent further complications [4].

**Table 1:** Case reports/series reporting osteo-articular infection associated with atopic eczema [modified from [9, 19]]

Case	Author - Year	Diagnosis	OA type	Age (Yr)	Gender	Reported atopic eczema severity	Micro-organism
1	Patel D; 2015 [4]	Septic arthritis and osteomyelitis - left shoulder	OAOM	8	F	Severe	MRSA
2	Sayaka I; 2013 [21]	Costo-chondral abscess	OA	20	M	NS	NS
3	Tsutsumi R; 2010 [25]	Cervical spondylitis spondylitis	OA	31	M	Severe	SA
4	Kitamura S; 2000 [8]	Septic arthritis - hip	OA	15	F	Recent flare	SA
5	Boiko S; 1988 [5]	Osteomyelitis - distal phalanx	OM	3	M	Severe	MRSA, Strep
6	Boiko S; 1988 [5]	Osteomyelitis - distal phalanx	OM	4	F	Severe	MSSA, Strep
7	Boiko S; 1988 [5]	Osteomyelitis - nail plate	OM	2	F	Severe	MSSA, Strep
8	Sharma A; 1997 [2]	Osteomyelitis - fibula	OM	4	M	Severe	MSSA
9	Nassif A; 1994 [1]	Septic bursitis - mid-tibial, olecranon	OA	60	M	Severe	MRSA
10	Kusunoki T; 2015 [19]	Right hip osteo-arthritis	OA	0.25	M	Severe	MSSA
11	Kusunoki T; 2015 [19]	Left hip oste-arthritis	OA	11	M	Recent flare	NS
12	Numazaki H; 2017 [22]	*Tibial osteomyelitis (complication of ACL reconstruction)	OM	24	M	Severe	MSSA
13	Ohno et al.; 2000 [19]	Right sacroiliac	OA	13	M	Moderate-severe	MSSA
14	Ueda et al.; 2001 [19]	Left hip oste-arthritis	OA	3	M	Unknown	SA
15	Ono et al.; 2003 [19]	Knee SA	OA	Infant	Unknown	Moderate-severe	SA
16	Hidaka et al.; 2004 [19]	Right knee	OA	5	M	Moderate-severe	MRSA
17	Yamagata et al.; 2004 [19]	Right hip and knee SA	OA	12	M	Moderate-severe	MRSA
18	Kimura et al., 2005 [19]	Left hip	OA	0.25	F	Moderate-severe	MSSA
19	Nakamura & Fujioka; 2006 [26]	Left hip	OA	5	M	Moderate-severe	MSSA
20	Moriwaki et al.; 2006 [19]	Left sacroiliac	OA	21	F	Unknown	MSSA
21	Nakamura & Fujioka; 2006 [26]	Right knee and femur	OA	0.83	M	Moderate-severe	MRSA
22	Nakamura & Fujioka; 2006 [26]	Right hip	OA	0.5	F	Moderate-severe	MSSA
23	Nakamura & Fujioka; 2006 [26]	Right hip	OA	0.58	M	Moderate-severe	Strep
24	Hiyane et al.; 2007 [19]	Left hip	OA	11	M	Unknown	NS
25	Matsushita et al.; 2008 [19]	Left knee	OA	1	F	Mild	MSSA
26	Nagai et al.; 2008 [19]	Left tibia	OA	3	M	Mild	NS
27	Nagai et al.; 2008 [19]	Left femur	OA	3	M	Moderate-severe	MSSA
28	Nagai et al.; 2008 [19]	Left hip	OA	0.92	F	Moderate-severe	MSSA
29	Suzuki et al.; 2009 [19]	Right knee	OA	7	F	Moderate-severe	SA
30	Kinugasa et al.; 2009 [19]	Right hip	OA	23	F	Unknown	Strep
31	Hashi et al.; 2012 [27]	Right hip	OA	5	F	Moderate-severe	MRSA
32	Yamagata et al.; 2012 [19]	Right hip	OA	12	M	Moderate-severe	MRSA
33	Yasuda & Nisimatsu; 2012 [28]	Right sacroiliac	OA	15	M	Moderate-severe	Strep
34	Kyo; 2014 [23]	Knee	OA	13	M	NS	SA
35	Kyo; 2014 [23]	Knee	OA	27	M	NS	SA
36	Current case 1	Phalangeal osteomyelitis	OM	8	F	Mild	NS
37	Current case 2	Tibial osteomyelitis	OM	11	M	Severe	NS

*Abbreviations*: *OA* Osteoarthritis, *OM* Osteomyelitis, *MRSA* Methicillin Resistant *Staph. Aureus*; SA *Staph.Aureus*, *Strep* Streptococcus, *NS* Not specified

Here we present two unusual cases of osteomyelitis possibly resultant from secondarily infected atopic eczema in African children. These children were referred on the same day to our dermatology clinic from orthopaedic surgeons for the management of the underlying atopic eczema after initial admission for phalangeal and tibial osteomyelitis respectively. In addition, a narrative literature review of similar case presentations has also been carried out to describe the demographic and clinical features of osteo-articular infections associated with atopic eczema. The occurrence of the two presented cases in light of similar previous case reports may not be coincidental and calls upon clinicians to be aware of the potential complications of atopic eczema [4].

## Case presentation

### Case 1

An 8 year old, black female child was referred to the dermatology team from orthopaedic surgeons with a 3 month history of a swollen right index finger. On further enquiry, the patient was noted to be atopic with comorbid chronic asthma and atopic eczema. The child was being managed on aqueous cream baths, topical betamethasone cream, a non-sedating antihistamine – loratadine and liquid paraffin as an emulsifying ointment and an asthma medication pump. On the current dermatology consultation, the child’s caregiver mentioned that the child had been scratching the itchy right index finger. In the period prior to the presentation, the child’s finger got swollen and was painful prompting the hospital visit and subsequent admission. No history of phalangeal trauma or diabetes mellitus was elicited from the patient’s caregiver, which was also confirmed in subsequent tests. The child was HIV negative.

Examination revealed a swollen, mildly fluctuant index and middle phalanges with draining sinuses. There was an eczematous plaque with scaling and no lichenification. The fingers were tender and warm to palpation. No dysmorphic features were observed and the child’s body temperature was unremarkable at 37.2 °C. The X-ray findings were consistent with osteomyelitis and the child was admitted by the orthopaedic surgeons for intravenous antibiotics (amoxicillin/clavulanic acid, due to the unavailability of cloxacillin in our institution, which would be the drug of choice) to control the acute infection as surgical drainage was not warranted. The patient was discharged a week later to complete a 1 month oral antibiotic therapy course at home and to continue with her eczema medication. On review, the cellulitis had healed and the eczema lesions had been unmasked. The phalanges displayed the eczematous plaque with diffusely demarcated borders. No weeping or crusting was observed. Her initial calculated (Eczema Area and Severity Index) EASI score and severity levels on presentation were 2.00 and mild severity respectively using the classification proposed by Leshem et al. [10]. The patient is now being followed up in the dermatology out-patient clinic for her atopic eczema.

### Case 2

An 11 year old, black male child was seen as a referral to the dermatology clinic at Edendale hospital, Pietermaritzburg with an 8 month history of bilateral leg swelling. The lesions started as ‘small pimples’ which subsequently drained pus and the legs got swollen and painful. Further enquiry showed that the patient had developed an itchy rash on both legs 10 months prior to the current presentation. No medical care had been sought, but the patient was applying a topical, traditional medicine to the legs with no relief of the symptoms. A history of trauma to the right leg about 5 months prior to the current presentation was noted in the patient’s history, but the other details were sketchily recalled by the patient. The rest of his past medical history was unremarkable with no previous history of atopy or diabetes mellitus.

Confluent, weeping plaques with diffuse edges and post-inflammatory hyperpigmentation were observed together with excoriations and a chronic draining sinus on the right leg. The left leg was swollen and erythematous, but had no sinus. The legs were tender on palpation, however there was full range of motion on both the ankle and knee joints bilaterally with normal pedal pulses. On the day of admission, the patient was apyrexial and he remained apyrexial during the period under admission. The complete blood count showed an unremarkable leucocyte count, 8.65 (4–10) cells/mm^3^ and a slight decrease in the haemoglobin count of 11.8 g/dl (12–15 g/dl). There was a marked increase in the markers of inflammation with an Erythrocyte Sedimentation Rate (ESR) of 45 (0–10) mm/hour, C Reactive Protein (CRP) 30 (0–5) mg/L and a platelet count of 500 (150–400) cells/mm^3^. The X-ray findings were consistent with chronic osteomyelitis of the right tibia, the left tibia X-rays were normal. The patient was HIV negative. However no pus swab was recorded in the patient’s charts. His initial calculated EASI score and severity levels on presentation were 9.60 and moderate severity respectively [10].

The patient was initially treated by orthopaedic surgeons as an acute-on-chronic osteomyelitis and intravenous amoxicillin/clavulanic acid was administered to manage the acute sepsis together with paracetamol as analgesia. On review by the dermatology team, the diagnosis was modified to chronic osteomyelitis secondary to infection with underlying chronic eczema. Potassium permanganate baths, topical betamethasone, silver-sulphur diazine, an emulsifying ointment and a non-sedating antihistamine – loratadine were added to his medical management. The patient was subsequently discharged to complete a course of rifampicin and trimethoprim/sulfamethoxazole, (orthopaedics current chronic osteomyelitis protocol for older children/adult patients) and was to be reviewed in both the dermatology and orthopaedic out-patient clinics. Further orthopaedic management has consisted of monthly clinic reviews, chronic suppressive antibiotics and sequestrectomy might be considered, should it be needed.

## Discussion and conclusions

In this case series, we have presented two unusual cases of osteomyelitis associated with severely infected atopic eczema in HIV uninfected children. The cases represent two of the three generally accepted mechanisms of osteomyelitis infection – contiguous spread and haematogenous spread [11]. The first case developed possibly after direct infection of the distal phalangeal bone due to an overlying septic eczema focus. The second case possibly had haematogenous spread to the tibial metaphysis. This was secondary to a bacteraemia resultant from the septic eczema, which then settled in the metaphysis as commonly happens in immature long bones [12, 13]. Both children had habitual, excessive scratching of their dry, fissured skin in poorly or untreated eczema respectively. In addition, there were no other apparent sources of infection in both cases.

The phalangeal osteomyelitis case is similar to three cases reported by Boiko et al. [5]. There was insidious onset of radiologically confirmed osteomyelitis without any associated fever or ESR elevation. This signified a localized infection as opposed to the second case which presented with tibial osteomyelitis associated with a raised ESR. The raised ESR in the second case indicates haematogenous spread, possibly of invasive staphylococcal infection presenting as osteomyelitis [5]. Even though no bacterial cultures were done prior to initiation of antibiotic therapy in both cases, we strongly suspect that *Staphylococcus aureus* was the causative organism. The lack of bacterial culture results may be a major limitation to our study. However, staphylococcal bacterial colonization has been shown to be more common in atopic eczematous skin compared to normal skin [14].

Furthermore, it has been postulated that recurrent bacterial and viral infections often complicate atopic eczema possibly due to an interplay between staphylococcal enterotoxins (super-antigens), cutaneous barrier defects and the dysfunctional cutaneous innate immune system [15]. The latter is characterized by increased skin pH and decreased antimicrobial peptides – human β defensins and cathelicidins coupled to increased CD4^+^ Th2 cytokines - IL-4 and IL-13 [15, 16]. Decreases in antimicrobial proteins are caused by the skewing of the lymphoctic response towards the CD4^+^ Th2 direction with increased cytokines IL-4 and IL-13 resultant from increased keratinocyte derived thymic stromal lymphoprotein expression in atopic eczema [15]. Additionally, the relapsing, itchy chronic inflammation of atopic eczema is also perpetuated by the reduced staphylococcal bacterial inhibition and low “natural moisturizing factor” (NMF) resultant from the lack-of-function filaggrin gene defects [15, 17]. The reduced NMF exacerbates atopic eczema due to the dry skin aggravating and perpetuating the “itch – scratch” cycle [15, 18]. Chronic scratching of the itchy skin potentially worsens the already weak cutaneous barrier, thereby facilitating the entry of allergens and pathogens into the skin [4, 19]. Consequently, cutaneous and extra-cutaneous infections occur with higher incidence in atopic eczema patients compared to non-atopic eczema patients [20]. Eczema herperticum, erysipelas, impetigo, cellulitis, otitis media and streptococcal throat infection have been widely documented in patients with atopic eczema [15, 17, 20].

However, clinicians need to keep a high index of suspicion for invasive staphylococcal infection especially in toxaemic eczematous patients [8, 16]. Systemic infections such as osteo-articular infections, infective endocarditis and pneumonia have rarely been diagnosed in association with eczema [1, 2, 5, 9, 16]. Overall, a total of at least 35 case reports of invasive osteo-articular infection have been published in association with atopic eczema including 6 case reports first collated in 2005 by Benenson et al. [9]. Most of these retrieved case reports, 30 (85.71%) have been reported in Japanese patients with the remainder described in other settings. Twenty nine (29) out of the 35 case reports were on septic arthritis whilst 5 were on osteomyelitis and 1 case report had both osteomyelitis and septic arthritis [4, 9, 19, 21–23]. Septic-arthritis mostly involved the hip joint 14 (48.28%) followed by the knee joint 8 (27.59%) Table 1.

The majority of the osteo-articular cases, 28 (80.0%) were observed in paediatric patients below 18 years of age whilst the remainder was mostly in young adults. The median and average ages of the patients described in the case reports were 6 (IQR; 2.75:15.0) years and 10.72 ± 12.20 years respectively with a 22:12 M; F ratio. The mode of the presumed severity level of the atopic eczema on presentation with osteo-articular disease was moderate – severe disease. *Staphylococcus aureus* was cultured in 28 (80.0%) whilst *streptococcus* (*β haemolytic group B streptococcus* or *Streptococcus viridans*) was cultured in 6 (17.14%) of the retrieved case reports. Methicillin resistant and methicillin sensitive *Staphylococcus aureus* were documented in 8 (22.86%) and 13 (37.14%) case reports of osteo-articular infection respectively. Both *Staphylococcus aureus* (all MRSA) and *streptococcus* were both cultured in 3 (8.57%) whilst the culture result was unknown in 4 (11.43%) of the case reports.

Japanese individuals predominated in the retrieved case reports [19] possibly because of the increased use of sensitive Magnetic Resonance Imaging (MRI) and bone scans [23, 24] for patient work-up in Japan compared to Africa for instance. Nevertheless, a significant number were also observed in other populations and were associated with osteomyelitis. This underscores the need for aggressive management of the underlying atopic eczema and a high index of suspicion for osteo-articular infections as they can cause growth disturbances, deformities and even death [24]. This is especially so in patients who present with a persistent fever, an elevated ESR and/or moderate-severe or flaring eczema as there might be secondary staphylococcal or streptococcal infection of the eczematous lesions sometimes with subsequent systemic spread [4, 9, 17].. Early and appropriate management of the systemic infections with intravenous antibiotics [23] may reduce the indirect health costs associated with the potential atopic eczema associated systemic infection.

## Data Availability

The datasets used and/or analysed during the current study are available from the corresponding author on reasonable request.

## Abbreviations

CD: Cluster of Differentiation
CRP: C Reactive Protein
ESR: Erythrocyte Sedimentation Rate
HIV: Human Immunodeficiency Virus
NMF: Natural moisturizing factor
